# Children perpetuate competence-based inequality when they help peers

**DOI:** 10.1038/s41539-023-00192-9

**Published:** 2023-09-20

**Authors:** Jellie Sierksma

**Affiliations:** Department of Developmental Psychology, Utrecht University, Utrecht, The Netherlands

**Keywords:** Human behaviour, Psychology

## Abstract

Exchanges of help between children are common and often have positive consequences. But not all help is equally beneficial, for example because some help does not provide an opportunity to practice and develop skills. Here I examine whether young children might perpetuate competence-based inequality by providing incompetent peers with less opportunity to practice and improve their skills compared to competent peers. Study 1 (*N* = 253, 6–9 years) shows that young children understand not all help is equally beneficial: Children think that peers who receive empowerment (hints) vs. non-empowerment (correct answers) help can learn more. Study 2 (*N* = 80) and 3 (*N* = 41) then assessed children’s (7–9 years) actual helping behavior in a lab-based experiment. Through a cover story, participants were introduced to two unknown, same-age children whom they later overheard were either good or not good at solving puzzles (Study 2) or math (Study 3). Subsequently, participants got to help both of them with a puzzle-quiz (Study 2) or a math-quiz (Study 3) by providing either empowerment or non-empowerment when they asked for help. Across both studies, children were more likely to provide empowerment help to competent peers, and non-empowerment help to incompetent peers. This work suggests that when young children perceive differences in competence (e.g., based on stereotypes), they contribute to maintaining the status quo by providing the most vulnerable students, that would profit the most from improving their skills, less opportunity to do so.

## Introduction

Disparities in educational performance related to socio-economic and immigrant background take root at an early age and widen throughout development^1^. Research on the factors that contribute to educational inequality often focuses on the role of schools, teachers, and parents^2–4^. However, how children themselves think about and behave towards their classmates might also influence the extensive barriers that some children face at school. The current research sheds light on one aspect of peer interaction that is very common at school, namely peer-to-peer helping^5–7^. Specifically, three preregistered studies were conducted that examine how children (6–9 years) help competent and incompetent peers. A central aim of this work is to understand whether young children might perpetuate competence-based inequality by providing incompetent peers with less opportunity to practice and improve their skills. Understanding how social processes impact inequality between children is crucial to create optimal circumstances for children’s development and learning. Such insight could, for example, inform how to best structure educational practices that involve cooperative and collaborative learning^8,9^, to make sure *all* children profit from the education they receive regardless of their background.

Children often help each other. Helping happens informally, for example, when someone has trouble tying their shoelaces or does not understand an assignment at school. Peer-to-peer helping is also a formal strategy at school that teachers implement because it is assumed to have benefits over and above individual or independent learning^5,6^. In practice this means that teachers create pairs or assign children to small groups, and they then work on specific assignments (i.e., collaborative learning). Sometimes this is also accompanied by assigning children specific roles (e.g., one person is a tutor). During collaborative learning exercises children thus often work together in pursuit of a shared goal^5^. Although unstructured peer-to-peer-helping and collaboration are also common^7^. Peer-to-peer helping and collaborative learning are assumed to have benefits because children help each other learn and also learn themselves when doing so.

The exchange of help undeniably often leads to positive outcomes and is particularly beneficial when it involves teaching others strategies and providing them with experience of solving challenges on their own. Such so-called empowerment help enables the development of skills and leads to improvement in performance (e.g.,^10–14^). Not all help is equally beneficial, however. For example, when children work on an assignment their classmates might not teach them strategies but rather provide them with the correct answer. Such help alleviates the child’s need in the short run but does not provide an opportunity to practice and improve. This type of help, defined here as non-empowerment help, is assumed to be less beneficial for recipients because it does not allow for improving skills and undermines feelings of autonomy and competence in recipients^15–18^.

Research suggests that adults often differentiate in the type of help they provide (e.g.,^10,17,19–22^). One prominent consideration in the decision to provide different types of help is someone’s perceived competence or status. Specifically, adults provide more empowerment help to those they believe are competent or higher in status, but more non-empowerment help to incompetent or lower status recipients^21,23,24^. Providing hints and strategies to solve challenges independently is probably seen as more effective when people believe others are sufficiently competent to use the help provided. When someone is perceived as incompetent, providing non-empowerment help might seem better. Although providing non-empowerment help to those perceived to be lower in competence might be well intentioned, this could lead to the perpetuation of competence-based inequality because it hampers the development of skills for those that might need it the most.

How children perceive and distribute empowerment and non-empowerment help is not clear. However, in the classroom children have ample opportunity to make inferences about their classmates’ competencies. Children probably track whether their classmates have trouble with tasks or make errors^25,26^. Within-classroom differentiation practices—also called ability grouping—also provide a rich set of cues about classmates’ competencies. Children, for example, observe which children in their classroom receive additional instruction or time, are assigned schoolbooks that offer additional challenge or which children get to participate in honor programs^3,27–30^.

Children’s perceptions of competence are, like those of adults^31,32^, also influenced by societal stereotypes. That is, from a young age onward children start to apply the stereotype that people belonging to disadvantaged groups (i.e., low SES, non-Western migration background) have lower intellectual ability. For example, White American children sometimes think that White people are smarter than Black people^33,34^. Children as young as 4 years also see poor peers as less competent than wealthy peers^35,36^ and assume native-accented speakers are more competent than people who speak with non-native accents^37^. Early emerging societal stereotypes likely thus also impact children’s perception of their classmates’ competencies.

Perceptions of competence can have an enduring impact on children’s social cognition and behavior. Children are more likely to trust people who are competent in providing factual and accurate information^38,39^. In one study, for example, 3-and-4-year-olds overheard that some people were accurate in naming familiar objects and others were not. Children trusted the accurate informants more immediately afterwards but also 1 week later^39^. Exposure to information about competency can also change children’s pre-existing notions about competence. For example, while children think that adults are more competent than peers, they change their mind when they learn the opposite is true^40^. Taken together, there is ample evidence that from a young age onward children infer people’s competence from different sources and that inferences about competence impact how they see and behave towards others.

There is also some evidence that children consider other people’s competence when they help them. In one study, 4-to-8-year-olds got to help other children who were either good at solving puzzles or sports or not so good at it^41^. Results showed that from 5 years onward, children provided more help to children that were incompetent compared to competent. In another study^42^ older children’s helping was influenced by ethnic stereotypes about competence. Specifically, children (10–13 years) helped ethnic out-group children more when they endorsed the stereotype that the out-group was less smart (suggesting they needed more help). These studies thus suggest that children provide more help to those they perceive as less competent, presumably because they assume that people lower in competence need more help^43^.

Research has not yet addressed, to my knowledge, whether children also help peers they perceive as competent and incompetent others *differently*. In general, developmental research on helping predominantly focuses on binary or graded differences in children’s prosocial behavior. That is, studies examine whether (yes vs. no) and how much or how long it takes children to start helping others see ^44,45^. Given that not all help is equally beneficial and that providing help can also have negative consequences it is crucial to better understand *how* children help others.

The three preregistered studies presented here aim to understand whether children might perpetuate competence-based inequality by providing incompetent children with less opportunities to practice compared to competent children. Study 1 (6–9 years) was set out to understand when children understand that not all help is equal. At what age do children think, like adults, that children who receive empowerment help (hints) versus non-empowerment (correct answers) are smarter and will learn more? Study 2 and 3 focused on 7-to-9-year-old children and assessed children’s actual helping behavior in a highly controlled experimental lab-based study to establish causal factors that drive children’s differentiated helping. As such, these studies provide a stepping stone for future studies assessing helping in a more naturalistic setting in the classroom. In both studies we used a cover story: Participants were introduced to two unknown, same-age children whom they later overheard were either competent or not so competent at a task. Subsequently, participants got to help both of them with the task by providing either hints (i.e., empowerment help) or correct answers (i.e., non-empowerment help) when they asked for help.

Children tested were 6–9 years of age because children this age frequently help each other and information about competence has an enduring impact on their social cognition and behavior^39^. From an intervention perspective it is also important to focus on young children as educational inequality emerges early^1^ and children’s views of competence and groups are probably more malleable early in life^46^. Non-empowerment and empowerment help can take different forms. Here, we operationalized non-empowerment help as correct answers and empowerment help as providing hints (i.e., hints that teach children strategies). We did so because these types of help are likely familiar to children, common in the classroom, and often studied in previous research^21,22,47^.

## Results and discussion

### Study 1

Children as young as 3 years of age perceive helping and sharing positively^48,49^ and their reasoning becomes more sophisticated with age^50–52^. There is some evidence that children from 7 year onward respond differently when they receive empowerment and non-empowerment help (but responses differed according to children’s gender^47^). However, no research to date examined whether young children understand that not all help is equally beneficial.

Study 1 therefore investigates the inferences children make when they observe that some people receive empowerment help and others receive non-empowerment help. Children saw animated videos about groups of children who worked on academic tasks (i.e., a word game, an art-project, a puzzle, based on earlier work^53^). After seeing that each group received a different kind of help (hints vs. correct answers), we asked children which group they thought was smarter or which group would learn more. Preregistered hypothesis stated that when people receive hints, children will perceive them as smarter and think they would learn more compared with people who receive the correct answer^21^. To explore developmental differences, children across a wide age-range (6–9 years) were included.

A mean score was computed across the three videos for each dependent variable. A *t*-test comparing children’s inferences to chance (0.50) showed that children more often picked the group that received a hint (vs. the correct answer) when asked who was smarter (*t*(133) = 5.21, *p* < .001, *d* = 0.45, 95% CI = [0.27, 0.63]) or which group would learn more (*t*(118) = 9.78, *p* < .001, *d* = 0.90, 95% CI = [0.68, 1.11]). This was true for all three tasks (i.e., word-game, puzzle and art-project; smart, all *p’s* < .04; learning, all *p’s* <.001). There were no gender differences.

To analyze the influence of children age, mixed logistic regression was conducted in R including a random intercept (note that logistic regressions were preregistered but in hindsight the random intercept is necessary to account for dependency in the data^54^). Results showed a main effect for age (in years, continuous variable) for children’s inferences about being smart (*β* = 0.66, SE = 0.14, *p* <.001 95% CI = [0.39, 0.97]) and learning (*β* = 0.76, SE = 0.22, *p* < .001, 95% CI = [0.34, 1.24]). Simple slope analyses showed that older children (1 SD above the mean) thought that children who received hints (vs. correct answers) were smarter (*β* = 1.40, SE = 0.23, *p* < .001, 95% CI = [0.99, 1.89]) and would learn more (*β* = 2.73, SE = 0.43, *p* < .001, 95% CI = [1.99, 3.71]). Younger children (1 SD below the mean) thought both groups were equally smart (*β* = 0.08, SE = 0.18, *p* = .66, 95% CI = [−0.28, 0.44]) but thought groups that received hints would learn more (*β* = 1.20, SE = 0.32, *p* < .001, 95% CI = [0.63, 1.96]).

Study 1 shows that 6-to-9-year-old children think that groups that receive hints will learn more than groups that receive correct answers. Moreover, with the exception of younger children (i.e., around 6 years), children also thought receiving hints was indicative of being smarter than receiving answers. The type of help received did not impact younger children’s inferences about smartness, however. Perhaps it is easier for young children to identify the immediate consequences of type of help received compared to making additional trait-level inferences, a possibility future research could address. Taken together these results suggest that certainly by 7 years of age children understand that not all help is equally beneficial.

### Study 2

Study 2 was designed to test whether children might perpetuate competence-based inequality by distributing different types of help to peers they perceive as competent or incompetent. Young children highly value fairness^55^. Moreover, older (10–12 years) children tend to also rectify inequality by, for example, providing more resources to disadvantaged groups^56,57^. At the same time, younger children often seem less inclined to rectify inequality^56–58^ and sometimes even perpetuate inequality, in particular when it concerns wealthy and poor recipients^59^. For example, in one study 4-to-11-year-olds perpetuated inequality by giving more cookies to targets that already got plenty^60^. Similarly, 4-and-5-year old children were more likely to give a bigger piece of chocolate, rather than a small one, to a higher status puppet than a lower status puppet^61^. Based on these findings and work with adults^21^ preregistered hypothesis stated that children will perpetuate competence-based inequality and provide more empowerment help (compared to non-empowerment help) to competent targets rather than incompetent targets.

Study 2 and 3 also explored the role of children’s mindsets about competence in their competence-based helping. One reason to provide empowerment to others is that they will improve their skills when they practice. However, practice will only lead to improvement when someone’s competence in that domain is (believed to be) malleable. Children might differ in whether they think other people can change their competencies^62–64^. When children endorse a fixed mindset, this entails that they perceive traits such as intelligence as not changeable. A growth mindset, in contrast, suggests that competence is seen malleable. People that endorse a growth mindset tend to focus on learning and exerting effort to improve skills and see failure as an opportunity to improve. Whereas, a fixed mindset is often associated with a tendency to avoid challenging tasks and exerting effort is perceived as revealing low competence^62,65,66^. There is a large literature documenting that children’s mindsets about competence have important consequences for their motivation and achievement in school. Less is known, however, about how mindsets influence children’s behavior toward others (but see refs. ^67,68^). Study 2 and 3 explore whether children provide incompetent others with more opportunity to practice and are thus less likely to perpetuate competence-based inequality, when they believe change is possible.

As a prerequisite for differentiated helping might be that children understand the implications of empowerment and non-empowerment help, Study 2 focuses on 7-to-9-year-old children. In addition to assessing children’s competence-based helping and their mindsets about competence, Study 2 also included a measure of how nice children thought each target was. As children sometimes like smart people better than less smart people^69^, this measure allowed us to rule out that differences in niceness drove children’s helping behavior. In addition, to further explore how children perceive hints and answers, we asked them whether others could learn more from hints or answers (i.e., replicating Study 1) and which type of help would make others happier.

#### Manipulation check

Children thought the competent target was smarter than the incompetent target, *t*(79) = 17.93, *p* < .001, *d* = 2.01, 95% CI = [1.62, 2.38], which indicates the competence manipulation was successful.

#### Influence of target competency on type of help

**Fig. 1:**
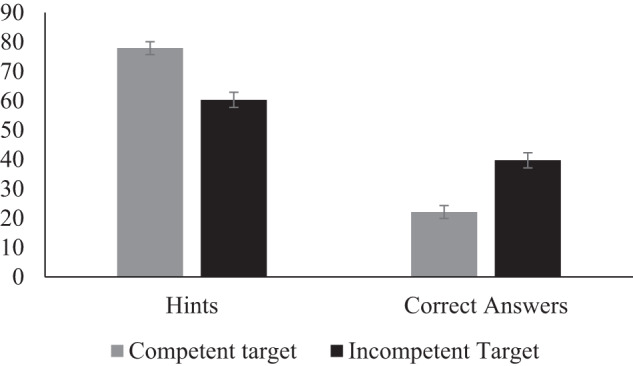
Percentage of times children provided hints and answers to competent and incompetent targets, Study 2. Note. Error bars represent standard error.

A significant intercept showed that children were more likely overall to provide hints compared to correct answers (*β* = 0.80, SE = 0.08, p < .001, 95% CI = [0.65, 0.96]; 68.4% hints, 31.6% correct answers). But there was also a significant main effect for competency (*β* = 0.37, SE = 0.08, *p* < .001, 95% CI = [0.22, 0.52]: Children’s tendency to provide more hints than correct answers, was stronger for competent compared to less competent targets (see Fig. 1). This is in line with the hypothesis that children provide less opportunity to practice to incompetent peers. No significant main effects or interactions with the contrast for competency were found for children’s age (main affect age: *β* = −0.07, SE = 0.11, *p* = .53, 95% CI = [−0.28, 0.15]; interaction age and competency: *β* = −0.11, SE = 0.10, *p* = .28, 95% CI = [−0.32, 0.09]) or their gender (main effect gender: *β* = 0.06, SE = 0.08, *p* = .47, 95% CI = [−0.22, 0.10]); interaction gender and competency: *β* = 0.08, SE = 0.08, *p* = .36, 95% CI = [−0.24, 0.08]).

#### Mindsets

Correlations between the four questions about mindset were low (*r* ranged from .03 to .23) and, as preregistered, analyses therefore first focused on the influence of each item separately (note that 1 child did not answer these questions and thus analyses include *n* = 79). For instability, there were no main or interaction effects (*p*’s ranged from .62 to .93) and for malleability there were also no effects (*p*’s ranged from .26 to .78). In addition, the influence of children’s mindset overall (mean score of four mindset questions = 2.72, SD = 0.47) was also not significant (main effect: *β* = 0.08, *SE* = 0.08, *p* = .33, 95% CI = [−0.08, 0.23]). interaction: *β* = 0.01, SE = 0.08, *p* = .88, 95% CI = [−0.14, 0.16].

Despite the absence of significant interactions for children’s mindsets with the type of help they provided, children’s answers to these questions revealed an interesting pattern. Specifically, when asked whether targets’ competency in the quizzes could change (instability question), children were more likely to think this was the case for incompetent targets compared with competent targets respectively, *M* = 3.05 (SD = 0.85), *M* = 2.28 (SD = 0.73), paired *t*-test (78) = 6.01, *p* < .001, *d* = 0.68, 95% CI = [0.43, 0.92]. Moreover, when asked what would happen when the targets would (not) practice (malleability question), children were more likely to expect that incompetent targets (*M* = 3.10, SD = 0.81) would change compared with competent targets (*M* = 2.43, SD = 0.83), *t*(78) = 6.06, *p* < .001, *d* = 0.68, 95% CI = [0.44, 0.93]. Overall, these results suggest children were more likely to endorse the idea that incompetent targets could change their skills compared to competent targets.

#### Niceness

Children thought that competent and incompetent targets were equally nice (respectively, *M* = 4.00, SD = 0.99, *M* = 3.88, SD = 1.12), *t*(79) = 0.96, *p* = .34, *d* = 0.11, 95% CI = [−0.11, 0.33]. How nice children thought competent targets (interaction with competency: *β* = −0.03, SE = 0.08, *p* = .70, 95% CI = [−0.21, 0.14]) or incompetent targets were did not influence their helping behavior (interaction with competency: *β* = −0.14, SE = 0.08, *p* = .11, 95% CI = [−0.30, 0.03]; note that the model for perceived niceness of incompetent targets were estimated without random components, as inclusion of random intercept resulted in singular fit).

#### Evaluation type of help

A higher proportion of children indicated that they expected others to be happier receiving an answer compared to receiving a hint (respectively, 72% vs. 28%, one child did not answer this question; binomial test *p* < .001). However, and in line with Study 1, almost all children thought that someone would learn more from receiving a hint (91%) than an answer (9 %, 1 child did not answer, binomial test *p* < .001). When we included children’s evaluation of the types of help in the logistic model (due to singular fit, logistic regression was conducted without random component for these moderators), results showed that children were more likely to provide hints when they expected this would make others happier than answers (*β* = −0.24, SE = 0.10, *p* = .01, 95% CI = [−0.44, −0.07]), but this expectations did not impact the type of help they provided to competent and incompetent targets (*β* = 0.09, SE = 0.09, *p* = .34, 95% CI = [0.09, 0.28]). The type of help children’s expected others to learn more from also did not influence their helping (main effect *β* = −0.14, SE = 0.14, *p* = .32, 95% CI = [−0.40, 0.14]; interaction with competence: *β* = 0.09, SE = 0.14, *p* = .50, 95% CI = [−0.17, 0.39]).

Study 2 provides a first demonstration that children provide different types of help depending on whether they perceive recipients as competent or incompetent. Specifically, children were more likely to provide empowerment help (vs. non-empowerment help) to those they perceived as competent rather than incompetent. Exploratory analyses further revealed that children expected others to be happier receiving correct answers, while acknowledging that hints would provide more opportunities for learning, similar to Study 1. In addition, it was not the case that children perceived one of the targets as nicer that the other and perceptions of niceness did not influence their helping. Children’s mindsets about the recipients competence also did not influence their helping. Taken together, these results thus suggest that children as young as 7 years, perpetuate competence-based inequality through their helping.

### Study 3

In Study 2, children perpetuated inequality but at the same time endorsed the idea that incompetency is malleable. Specifically, children thought that being incompetent was less stable than being competent. And children assumed that if an incompetent peer would practice their skills, their skills would change more compared to a competent peer who did not practice. These results suggest that children do endorse the idea that incompetency can be overcome. So why did they not help incompetent children accordingly and provide them with more opportunities to practice that already competent peers? Results of Study 1 and 2 suggest that this is not because children this age fail to understand the diverging implications of providing hints versus correct answers. One reason children might have been more prone to provide correct answers to incompetent children is that they did not associate “playing a puzzle quiz” with something people are able to get better at. Perhaps playing a puzzle quiz is associated with “fun” and “luck” rather than practicing and learning. Study 3 was designed to get some purchase on that question by having children help with a task that is more closely related to the educational context, namely a math quiz.

Children this age are familiar with learning math at school and typically practice with math, including in peer-to-peer-helping contexts^70^. In addition, math is often perceived as a challenging topic^71,72^. As such, a math test might make learning and practicing more salient to children. To further amplify that the task was about learning and practice, in Study 3, it was also emphasized that the first round was a practice round to help children do better in round 2.

Study 2 showed that mindsets did not impact children’s helping. However, we only assessed children’s mindsets about competencies of peers and used a novel measure. Previous research suggest that children’s motivational frameworks are related to math achievement as early as first grade^73^. So perhaps mindsets about children’s own abilities are more influential in their helping. Study 3 therefore included validated measures to assess children’s mindsets about their *own* intelligence and ability in math.

#### Manipulation check

Similar to Study 2, the manipulation was successful because children perceived competent targets as smarter than incompetent targets, *t*(40) = 12.84, *p* < .001, *d* = 2.01, 95% CI = [1.47, 2.53].

#### Influence of target competency on type of help

**Fig. 2:**
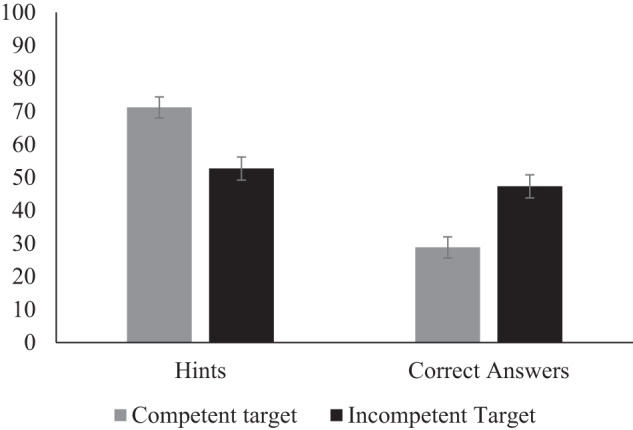
Proportion of times children provided hints and answers to competent and incompetent targets, Study 3. Note. Error bars represent standard error.

Preregistered analyses (logistic linear mixed effects model, including competency with a random slope and a random intercept) showed that children overall gave more hints than answers to targets (*β* = 0.54, SE = 0.13, *p* < .001, 95% CI = [0.28, 0.79]; 62% hints, 38% answers). As in Study 1, however, this tendency was moderated by target competency (*β* = 0.43, SE = 0.11, *p* < .001, 95% CI = [0.21, 0.63]; see Fig. 2). These results suggest that even when the task involved solving math problems and practice was emphasized children were less inclined to provide more hints than answers when someone was not competent. Helping behavior was not influenced by children’s age (interaction with competency *p* = 0.19) or gender (interaction with competency: *p* = .87; models only included random intercepts as the preregistered models did not converge or resulted in a singular fit).

#### Mindsets

The influence of mindsets was analyzed using logistic models with random intercept only as the full model resulted in singular fit. Children’s mindsets about other target’s abilities did not correlate strongly (ranging from *r* = 0.04 to *r* = −0.29) and therefore effects were assessed for each item separately first. There were no main or interaction effects for how stable children thought being competent (main effect: *p* = .64; interaction with competency: *p* =.17) or incompetent was *(*main effect: *p* = .06; interaction with competence: *p* = .06). And children’s endorsement of malleability also did not influence how they helped competent and incompetent targets (malleability competence, main effect *p* = .08, interaction *p* = .08; malleability incompetence, main effect *p* = .52, interaction *p* = .25). A mean score also did not influence the type of help children gave (main effect *p* = .66, interaction *p* = .16).

As in Study 2, children were more likely to think that being incompetent (*M* = 2.24, SD = 0.92) could change compared to being competent (*M* = 2.73, SD = 0.81), *t*(40) = 2.59, *p* = .013, *d* = 0.40, 95% CI = [0.08, 0.72]. And children also thought that being incompetent (*M* = 3.20, SD = 0.90) was more malleable that being competent (*M* = 2.46, SD = 0.87), *t*(40) = −3.40, *p* = .002, *d* = −0.53, 95% CI = [−0.86, −0.20]. Children thus again were more likely to endorse the possibility for change for incompetent peers compared to competent peers.

No main or interaction effects were found for children’s mindset about their own intelligence (main effect: *β* = −0.12, SE = 0.12, *p* = .32, 95% CI = [−0.37, 0.12]; interaction: *β* = 0.10, SE = 0.10, *p* = .35, 95% CI = [−0.30, 0.11]). For math there was no main effect (*β* = −0.04, SE = 0.13, *p* = .76, 95% CI = [−0.30, 0.22]) but the interaction with competence was significant (*β* = −0.22, SE = 0.11, *p* = .049, 95% CI = [−0.43, −0.002]). Simple slope analyses showed that when children endorsed a growth mindset about their math abilities (i.e., 1 SD below the mean), their tendency to provide more hints was stronger for competent compared to incompetent targets (*β* = 0.62, SE = 0.15, *p* < .001, 95% CI = [0.33, 0.93]). Whereas, when children endorsed a fixed mindset (i.e., 1 SD above the mean), they did not differentiate between competent and incompetent targets (*β* = 0.19, SE = 0.15, *p* = .21, 95% CI = [0.33, 0.93]). This pattern is opposite of what was expected. However, the interaction was barely significant and should thus be interpreted with caution.

#### Niceness

Children thought competent (*M* = 3.76, SD = 0.99) and incompetent (*M* = 3.85, SD = 0.96) targets were equally nice, *t*(40) = −0.64, *p* = .52, *d* = −0.10, 95% CI = [−0.41, 0.21]. And perceptions of niceness did not influence how they helped them (niceness competent target, main effect *p* = .75, interaction *p=* .81; niceness incompetent target, main effect *p* = .51, interaction *p* = .26).

#### Evaluation type of help

Children expected others to be somewhat happier receiving answers (61%) compared to receiving hints, but not significantly so (binomial test, *p* = .211). Similar to Study 1 and 2, children did think others would learn more from receiving hints (92.2 %) compared to answers (binomial test *p* < .001). Children’s evaluations, however, did not influence their helping behavior expectations of happiness, main effect *p* = .14, interaction with competence, *p* = .46; expectation about learning (main effect, *p* = .38, interaction with competence, *p* = .50).

Study 3 shows that children’s tendency to provide more empowerment help than non-empowerment is less pronounced when they are helping incompetent peers. This tendency to perpetuate competence-based inequality emerged despite the fact that the task was explicitly about practice and learning.

## General discussion

Children often help each other, and exchanges of help play a central role in their education^6^. The current research aimed to shed light on how children’s helping behavior can reinforce competence-based inequality between peers. Results show that children as young as 7 years understand that not all help is equally beneficial. Moreover, when children helped others they maintained the status quo rather than improving competence-based inequality: They provided less opportunities for improvement to those they perceive as incompetent. Helping others improve, although children endorse that this is possible, does not seem to be at the forefront of their helping behavior in situations that involve differences in competence.

### Not all help is equal

A general assumption underlying much developmental research on prosocial behavior is that helping others predominantly leads to positive outcomes and is something we should stimulate^74,75^. However, across various disciplines, research indicates that prosocial exchanges can also negatively affect learning^12,22^, self-perception^16^ and social bonds^17^. The current research, shows that young children understand that not all help is equally beneficial. Specifically, in Study 1 6-to-9-year-old children thought that receiving empowerment help, as opposed to non-empowerment help, meant that recipients would learn more. Older children in this study also indicated that groups that received empowerment help were smarter than groups that received non-empowerment help. The findings for perceptions of learning were replicated in Study 2: Children (7–9 years) said that other children would learn more from receiving empowerment help. Overall, these results suggest that children, at least by age six and possibly at younger ages, understand that not all help has equally positive outcomes.

Study 2 and 3 further show that young children not only understand the difference between empowerment and non-empowerment help but also help peers differently. In both studies, children (7–9 years) provided help to peers playing a quiz and children in general provided more empowerment help than non-empowerment to peers. However, overhearing information about a peer’s competency changed the type of help children provided. Specifically, children were less likely to provide more empowerment (vs. non-empowerment) help when peers were perceived as incompetent compared to competent. This tendency was found both when children provided help with a puzzle quiz (Study 2) and a math quiz for which we emphasized learning and practice (Study 3). The impact of perceived competency on the type of help children provided was thus not due to the type of task children provided help with. Taken together, this research suggests children as young as seven years provide less opportunities for improvement to those they perceive as incompetent.

When children help those they perceive as incompetent in less empowering ways the implications are manifold. Such differentiation obviously provides less opportunities for improvement to those that already struggle the most. There is also evidence that receiving non-empowerment help leads recipients to think less well about themselves^16^ and bystanders also think more negatively about those recipients^21,53^. Which means that biases in helping not only hamper the development of skills but might also contribute to negative (self)perceptions of recipients of help. These negative implications of differentiated helping likely impact children belonging to disadvantaged groups in particular: Children at a young age start to endorse a deep-rooted societal stereotype that disadvantaged groups are less competent^33–37^ and ample research shows that children with immigrant and lower socio-economic backgrounds are overrepresented in lower ability groups^76–80^.

Taken together this research points to the possibility of a self-sustaining circle, that starts early in life, in which the most vulnerable students that would profit the most from improving their skills receive less opportunity to do so from their peers. Peer-to-peer helping and collaboration might thus not only facilitate educational success but could also reinforce competence-based disparities.

### Why do children perpetuate competence-based inequality?

It is important to better understand why children helped competent and incompetent peers differently. The current research shows that children’s differentiated helping is not driven by how nice children thought competent or incompetent targets were. That is, across both studies children perceived incompetent and competent peers as equally nice and their perceptions of niceness also did not influence the type of help they provided.

Study 2 and 3 also explored whether children’s mindset influenced their helping behavior. Children who endorse learning and practice (i.e., a growth mindset) might be more inclined to provide empowerment help to others, especially when they will benefit from improvement (i.e., are lower in competence). Study 2 and 3 therefore assessed children’s mindset about ability of the recipients of help (using vignettes) and Study 3 also assessed mindsets about children’s own ability (using traditional survey questions). Overall, however, there was little indication that children’s mindsets had an impact on the type of help they provided. With the exception of one finding that emerged in Study 3, showing that when children endorsed a growth mindsets they actually perpetuated competence inequality more rather than less (i.e., provided more hints to competent compared to incompetent peers). This finding was opposite of what was expected, however, and the effect was small and barely significant and should thus be interpreted with caution.

At the same time, the assessment of children’s mindsets about the competencies of peers (Study 2 and 3) revealed an interesting pattern. Across the two studies, children indicated that incompetency is less stable and more malleable that being competent. This pattern of results is in agreement with earlier work that showed that 4-to-6-year-old children thought unfamiliar peers were less likely to change their ability in math, spelling, and drawing when they were already competent compared to not competent^63^. As such, young children seem inclined to think that others who are not yet competent in a domain are able to change their skills. However, helping incompetent peers achieve that change does not seem to drive their helping behavior.

There are several possible reasons for why mindsets in these studies were not related to children’s helping behavior. For example, it is often assumed that stable mindsets develop later in life (10 years and older, see refs. ^63,66^) and thus mindsets are unlikely to drive young children’s helping behavior. However, most research on mindsets focused on older children (but see refs. ^73,81^) so we simply do not know much about mindset development early in life. But perhaps the current studies were also underpowered to detect the small effects mindsets often yield on children’s behavior^82^. Further research on the motivations driving children’s tendency to perpetuate competence-based inequality is crucial to better understand how and what could be successful ways to prevent it.

One possible mechanism that future research could address is that perhaps children perpetuated competence-based inequality because they felt sympathy or pity for incompetent peers. The Stereotype Content Model postulates that competence and warmth are central dimensions of social perception^83^. When adults perceive others as warm and incompetent this elicits feelings of pity and consequently a motivation to provide help (i.e., “paternalistic stereotype”)^31^. In the current work children perceived both peers as nice (i.e., warm) and might thus have felt, like adults, pity for children that were lower in competence. As a consequence, their helping might have been driven by the motivation to make the quiz easier for incompetent peers which led them to provide non-empowerment help to incompetent peers^42^. Such paternalistic tendencies are in line with earlier work showing paternalism in children as young as 5 years^84,85^. Future work could assess children’s emotional responses or ask children afterwards how they decided what help to give to understand if pity and sympathy drive differentiation in helping.

### Limitations and directions for future research

The current research provides important new insights into how helping between children takes form but is not without limitations. First, in the current work we used forced-choice measures and a within-subjects design in which children helped incompetent and competent targets at the same time. It will be important for future work to address how these design choices affected the results. For example, the design we used likely made differences between peers and types of help very salient to children. Effects might be smaller when such contrasts are not present (e.g., Likert-scales, between-subjects conditions)^86–88^. Similarly, we added descriptions to each type of help in our study to make sure the consequence of each type of help was clear and both were perceived positively. Future work test should test whether these specific definitions of hints vs. answers changed children’s helping behavior.

Further, the tightly controlled experimental set-up of this research has advantages in drawing causal relations but also leaves open questions about how children help in more ecologically valid circumstances. Future research should assess how children’s tendencies to perpetuate competence-based inequality play out in the classroom between peers that already know each other. Such work could also provide more insight into other forms of helping. In the current work, the type of help children could provide was restricted to hints and answers. But of course, children might also provide other types of non-empowerment and empowerment help (e.g., asking questions, taking over; see for example^89^) or might not want to help at all.

Examining perpetuation in the classroom is also an important next step because it would allow for measuring, rather than manipulating, children’s inferences about their classmates’ competencies and how these inferences impact their helping. Based on previous research, it is likely that children perceive classmates belonging to lower socio-economic and immigrant backgrounds as lower in competence^34,35,90^ and there is evidence that this can impact their prosocial behavior^42,91–93^ but we do not know whether it also affects the type of help children provide (see also ref. ^43^).

When teachers implement peer-to-peer teaching practices, they often assume learning from teaching is particularly powerful when lower competence children work with higher competence children (e.g.,ref. ^94^). It would be interesting for future research to assess how competency of the helper influences the type of help provided to peers that are perceived lower and higher in competence. In addition, peer-to-peer helping is assumed to foster learning and achievement. Future work could therefore also measure how well children do at the task they get help for. In sum, the current research suggest that prosocial exchanges might not always lead to positive outcomes and that it is crucially important to go beyond graded differences in prosociality and examine *how* children help others. The studies presented here provide a first step in doing so and gives rise to many intriguing new questions.

## Conclusion

The current research can contribute to our understanding of educational inequality. First, a promising solution to reduce gaps in educational inequality is to promote positive intergroup relations at an early age as these are expected to reduce prejudice and discrimination^95–97^. However, such calls often overlook that the prosocial relations between children can also have negative effects, which potentially worsen, rather than improve, inequality. Second, existing efforts to reduce educational inequality often focus on institutional practices and societal level infrastructures. A better understanding of how children themselves perpetuate inequality will create new pathways for creating circumstances in which we allow children to also be agents of change. The current research thus provides a much-needed child-centered perspective in transdisciplinary efforts to foster all children’s learning and thriving at school.

## Methods

All studies presented in this paper were approved by the ethical board of the university where the research was conducted (Study 1: VCWE-2019-120, Study 2: VCWE-2019-154, Study 3: 22-0310). For all participants signed parental consent was obtained and children provided assent for participation. All studies were programmed in Inquisit^98^ and children always worked independently. Specifically, in each study children were seated in front of a laptop, wore headphones and instructions were always provided via pre-recorded audio. For all studies participants received a certificate afterwards that said they contributed to science. All tests reported in the manuscript are two-tailed.

### Study 1

#### Participants

The preregistered target sample size was 192 children. Data collection, however, took place in a science museum (Science Live at NEMO Museum Amsterdam) for a set time of 2 weeks in 2019 and we included all 6-to-9-year-old-children that wanted to participate during that time. Our final sample consisted of 253 children between 6 and 9 years (see Table 1 for demographic data).

**Table 1:** Overview of demographic data for each study.

	Study 1	Study 2	Study 3
Mean age (SD)	7.73 (1.08)^a^	8.20 (0.79)^d^	8.11 (0.86)
Gender	49% boys	56.7% boys	61% boys
	47.4% girls^b^	36.3% girls	39% girls
Ethnicity	85.77% Dutch^c^	75% Dutch^e^	80.5% Dutch^f^
*Gross annual household income*^*g*^			
>35.000	-	10%	7.3%
35.000-70.500	-	17.5%	29.3%
<70.500	-	31.3%	36.6%
Did not report	-	41.3%	26.8%
*Education caretaker 1*			
High-school or equivalent	-	27.5%	14.6%
Bachelor and/or master degree	-	53.8%	78%
Did not report	-	10%	7.3%
*Education caretaker 2*			
High-school or equivalent	-	30%	14.6%
Bachelor and/or master degree	-	53.8%	73.2%
Did not report	-	10%	12.2%

Demographic information provided by parents.

^a^8 did not report.

^b^9 did not report.

^c^8 did not report.

^d^9 did not report.

^e^16 did not report.

^f^4 did not report.

^g^Modal household income is EUR 75.200 in the Netherlands^112^.

#### Procedure

Children watched three animated videos. After each video they answered one question by clicking with the mouse on their answer. Testing sessions lasted approximately 10 min.

#### Videos

Videos were based on earlier research and created using Vyond (see OSF for sample videos and see ref. ^53^ for an extensive description of the videos). Videos had the exact same structure and lasted approximately 1 min and 30 s. In each video, children saw two groups of animated children who worked on solving a puzzle, a word-game or an art-project. Then an adult expert walked up to one of the groups and said (for the video about puzzles) “I see you are doing a puzzle.” The expert then walked toward the group’s table and said “I will help you. I’ll give you a hint” and stood next to the group’s table. After a short fade out, the expert then walked over to the other group and said, “I see you are doing a puzzle”, walked toward the group’s table and said, “I will help you. I’ll give you the correct answer,” and stood next to the group’s table.

Within each group, characters were mixed in terms of racial group (each group consisted of a White, Black, Asian and Latino character) and gender (2 boys and 2 girls) but wore clothes of the same color (i.e., green vs. red, blue vs. yellow, orange vs. purple). The adult expert was female or male and White, Black or Asian (randomized).

#### Inferences about competence and learning

**Fig. 3:**
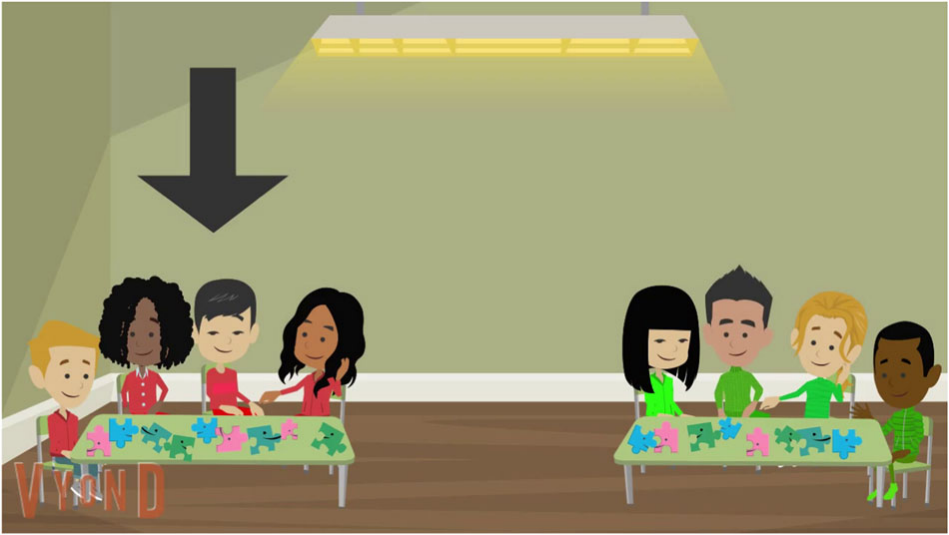
Last screen of the videos, Study 1.

Participants then saw a screen featuring just the two groups and the narrator said: “Now we would like to know how smart you think these groups are” (smart-condition) or “Now we would like to know how much you think these groups will learn from working on the puzzle/word-game/art-project” (learning-condition). Then a drop-down arrow pointed to the group on the left (see Fig. 3) and the narrator asked “do you think the [color] group is smarter/will learn more?”, and while a dropdown-arrow pointed at the group on the right, she said “Or do you think the [color] group is smarter/will learn more?”. Subsequently children saw a picture of each group and provided their answer by clicking on it. When children picked the group that received a hint this was coded “1” and when they picked the group receiving an answer this was coded “0”.

#### Design

The two dependent variables were measured between subjects because young children sometimes have a tendency to change their answer when asked a similar question twice^99^. Type of help (hints vs. answers) was manipulated within subjects. Across trials and participants, it was counterbalanced whether the expert first walked to the left or the right and whether groups received a hint or the correct answer. The order of the activities presented in the videos (puzzle, word game, art project) was also counterbalanced across participants.

### Study 2

#### Participants

Data collection took place in 2020 at schools (*n* = 22), after school daycares (*n* = 16) and in a science museum (*n* = 62). Data for six children was excluded because they were older than 9 years (2 10-year-olds, 4 11-year-olds; due to experimenter error). As preregistered, children were also excluded when they failed the memory check about targets’ competency during an earlier quiz (*n* = 14; pattern of results reported below does not change if excluded participants are included). The final sample (*N* = 80, 7–9 years) was larger than the preregistered sample of 64 because we collected data in a science museum and children that wanted to participate on the day we reached our final sample size were included. See Table 1 for additional demographic data.

#### Procedure and design

The entire procedure lasted ~15–20 min.

#### Introduction phase

First, children were told they were going to do a quiz that involved solving puzzles. We also told them the puzzles were similar (to assure that the help they would provide on one puzzle was also useful on the next). Next, we said: “You will be quizmaster! That is someone who is in charge and makes sure other people are able to play the quiz”. We then informed participants that they would be matched to two other children who would play the quiz and that these children were allowed to ask for help during the first round. We also said: “When they ask for help, you will see this on your screen. You then get to decide what kind of help you want to give”.

#### Practice phase

We showed participants two buttons and told them that they should press the yellow button to provide the correct answer and the orange button to provide hints. For hints, children were told these teach someone how to solve the puzzles themselves (i.e., in line with empowerment help and previous research^21,100^). Whereas, for answers we told them this would mean that someone could continue the quiz. We told children explicitly what these types of help meant, to make sure (lack of) differentiation in helping was not due to children interpreting the consequences of these types of help differentially. Moreover, to make sure children were aware it was o.k. to provide both types of help, each type of help was framed positively by highlighting benefits. Participants then practiced what button to press for each type of help and received feedback on their choice (“Yes, that’s right’ or ‘No, that’s not right, you should have pressed the yellow button!”).

#### Manipulation

To introduce the two recipients of help, children were told the “computer is now going to search for two other children” and they saw a timer counting down from 10. They were then introduced to each child by showing them a picture with a name (gender matched: Bas and Jan for boys, Sara and Julia for girls; pictures were pretested, see ref. ^101^). No information was provided about the age of the targets.

Competency of the targets was manipulated by letting children overhear an experimenter talk to the targets (see refs. ^102–106^ for evidence that overhearing messages impacts young children’s cognition and behavior). Specifically, participants were told that because they were assigned to the role of the quizmaster, they were allowed to listen to the instructions quiz-takers’ received. We also told them listening in was possible via the microphone on their computer. Participants then saw a picture of a big red button and the child whose instructions they were listening to. For each target they heard an experimenter say: “Okay, you can go and sit here. I’ll start the quiz. The quizmaster will tell you when to start. In the first round you are allowed to ask for help. Do you see the red button? If you want help, you can press it. Okay?”. Competency of the target was then manipulated (within subjects, counterbalanced order). For incompetent targets, they overheard the experimenter say “Last time it did not go so well eh? I heard the quiz was hard for you and you did not answer many questions correctly…”. For competent targets the experimenter said: “Last time it went really well eh? I heard the quiz was easy for you and you answered many questions correctly…”. The experimenter ended with saying they could start and wishing them good luck. Each audio clip was prerecorded by two research assistants (different from the narrator) and the order of whom delivered incompetent and competent information was counterbalanced.

Finally, participants were reminded that targets were now going to play the quiz. We remined participants that all the puzzles were similar and that the quiz-takers were only allowed to ask for help in the first round. This was important to make sure that children were aware that the first round was to practice and that practicing would be useful for the second round as they would learn how to answer the questions. We also emphasized that they were free to decide how to best help them and that quiz-takers could no longer ask for help in the second round.

#### Memory and manipulation check

We then checked whether children correctly remembered the overheard messages for each target, by asking: “Did [name target] think the quiz was hard or easy the last time?”. Children who failed to answer this question correctly, were excluded. A manipulation check was also included: “Do you think [name target] is smart or not so smart?”. Afterwards, children were told they could now start the quiz by pressing a button that said “START”.

### Measures

#### Helping behavior

**Fig. 4:**
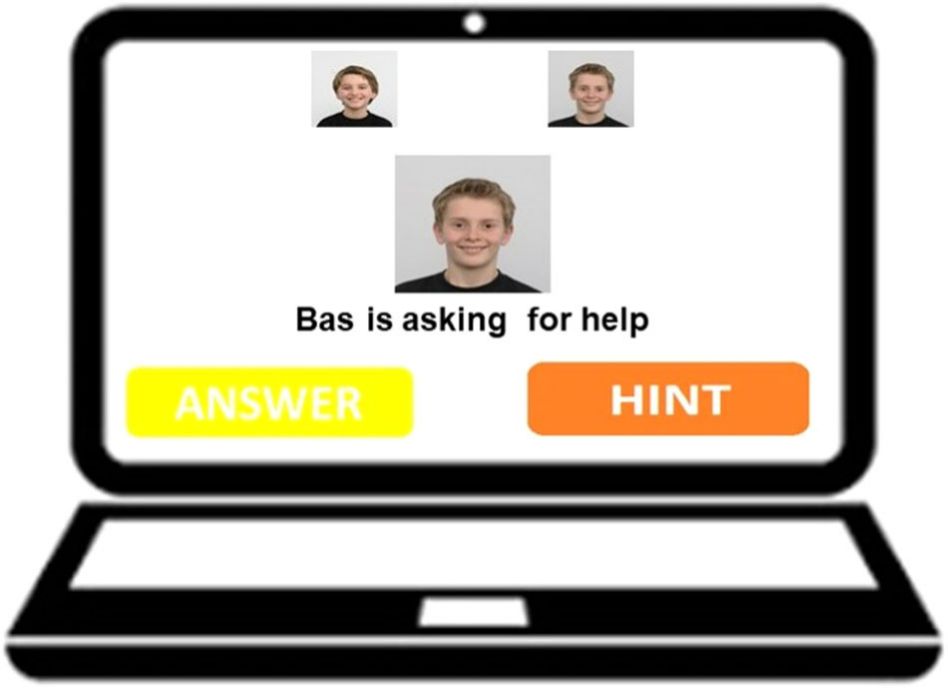
Set-up when providing help, Study 2 and 3. Note. Consent was obtained for publication of these photographs.

Children saw a screen with a photo of both targets and each of them asked for help five times alternately (which target asked for help first was counterbalanced). When this happened children heard the narrator say “[name target] is asking for help. You can decide how to help him/her” and a photo of the target was shown in the middle of the screen (see Fig. 4). Subsequently, children pressed the button for hints or answers and waited again until a message popped up that said a target asked for help. When targets had asked for help a total of ten times, children were told the first round of the quiz was finished and asked to answer some remaining questions we had.

#### Niceness

For each target, children were asked: “How nice do you think [name target] is ?” Answered on a 5-point scale ranging from “not nice at all” (1) to “very nice” (5).

#### Mindset

As no validated measure was available to assess children’s mindsets about *other* people’s abilities, we adapted the Growth Mindset Scale for Children, a measure specifically developed for use with younger children^63^. This measure assesses children’s mindsets by asking about people’s ability in terms of instability (“Will it always be this way?”) and malleability (i.e., how does (lack of) practice influence ability?) using short vignettes. In the original measure these vignettes are about unknown others doing math, spelling, and drawing, but here they were about the two targets children were matched to (e.g., Julia and Sara, or Jan and Bas) and children were only asked about the puzzle quiz targets did.

Children were always first asked about stability: “[name target] was not very good/very good (counterbalanced) at solving those puzzles. Will it always be this way?”. Children answered on a 4-point scale (absolutely (1) to absolutely not (4)).

Then malleability was assessed. For *competent* targets, children were told: “Imagine: [name target] is going to move and she goes to a new school. At this school they never practice with the type of puzzles from the quiz. [name target] thus never gets to do those puzzles again. Imagine that [name target] stays at this school a long time”. When targets were *incompetent*, participants were told the same story but now heard “At this school they practice a lot with the type of puzzles from the quiz. [name target] thus very often gets to do those puzzles.”. Participants were asked to indicate for each target ‘how well [name target] could do the puzzles when they left this school’, answered on a 4-point scale ranging from not well at all (1) to very well (4). The order of the malleability questions was counterbalanced.

#### Learning and appreciation of help

We asked children from which type of help others could learn more and which type of help might make others happier. For each question they could answer “Hint” or “Correct answer”.

#### Analyses

Data was analyzed in R (i.e., using lme4^107^). To examine whether competency of the recipient of help influences the type of help children provide, a logistic linear mixed-effects model was preregistered including a random intercept and random slope for competency. However, these models resulted in singular fit and therefore models were estimated with a random intercept only^108^. The main confirmatory model included one contrast comparing a competent (coded “1”) to an incompetent (coded “-1”) recipient of help. Exploratory analyses focused on the role of age, gender, mindsets, liking, and evaluation of hints and answers. All moderators are included as a continuous standardized score, except for children’s evaluation of help and their gender as this was a dichotomous variable (i.e., included as a contrast).

### Study 3

#### Participants

Sample size was estimated beforehand using summary-statistics-based power analysis for mixed-effects modeling of nested data^109^. We used the t-value for the main effect of a targets competency in study 2 (5.19) and study 2 sample size (*N* = 80) and then needed 34 participants to achieve 90% power (alpha is set at .05). Data collection took place in 2021–2022 at children’s homes (*n* = 17), after school day care (*n* = 6) and a science museum (*n* = 19). A total of 45 children participated because the last testing location was a science museum and, as preregistered, we included children who wanted to participate on the day we reached our target sample size. Four children were excluded (3 failed the memory check and 1 did not finish). The final sample consisted of 41 children between the age of 7 and 9 years (see Table 1 for demographics).

#### Design and procedure

The design and procedures were identical to Study 2 with the following exceptions. First, instead of a puzzle quiz, children were told they were going to do a math quiz that involved solving various math problems that were similar. Second, to emphasize the importance of learning we now explicitly told participants that the first round was to practice.

### Measures

#### Mindset

As in Study 2, mindset about other people’s abilities were assessed using vignettes. We also included a measure to assess how children view their own general intelligence (four items) and ability in math (four items). The latter was done using the revised self-theory scale^110,111^. Only the fixed mindset questions were used as these are less prone to social desirable answering^82^. We asked children, for example, how much they agreed with ‘To be honest, I don’t think I can really change how smart I am”. Children answered on a 4-point scale ranging from “not at all true” (1) to “very true” (4) and lower scores indicate a stronger growth mindset. To make sure the scale was appropriate for young children the word “intelligent” was changed to “smart” and a 4-point scale was used instead of a 6-point scale. See Supplementary materials for a list of all items. Reliability for both scales was excellent (Math, *α* = 0.83; General intelligence, *α* = 0.88).

#### Liking and Evaluation of Type of Help

These questions were the same as in Study 2.

### Supplementary information

SUPPLEMENTARY MATERIAL

nr-reporting-summary.pdf

## Data Availability

Studies were preregistered at Open Science Framework and data is posted there as well: https://osf.io/397tc/.
