# Supplementary material for: Children perpetuate competence-based inequality when they help peers
Source: NPJ Sci Learn. 2023 Sep 20;8:41. doi: 10.1038/s41539-023-00192-9 (PMC10511518; doi:10.1038/s41539-023-00192-9)
Supplement: Supplementary file 1 — SUPPLEMENTARY MATERIAL [file 41539_2023_192_MOESM1_ESM.pdf]

Supplementary Methods

Study 3: Mindset questionnaires

General intelligence

To be honest, I don't think I can really change how smart I am

I don’t think it can do much to change how smart I am

How smart I am is something about me which I cannot really change

I can learn new things, but I cannot really change how smart I am.

Math

To be honest, I don't think I can really change how good I am at math

I don’t think it can do much to change good I am at math

How good I am at math is something about me which I cannot really change

I can learn new things, but I cannot really change how good I am at math
