# Supplementary material for: Children perpetuate competence-based inequality when they help peers
Source: NPJ Sci Learn. 2023 Sep 20;8:41. doi: 10.1038/s41539-023-00192-9 (PMC10511518; doi:10.1038/s41539-023-00192-9)
Supplement: Supplementary file 2 — nr-reporting-summary.pdf [file 41539_2023_192_MOESM2_ESM.pdf]

nature portfolio

Corresponding author(s): Jellie Sierksma

Last updated by author(s): Sep 6, 2023

Reporting Summary

Nature Portfolio wishes to improve the reproducibility of the work that we publish. This form provides structure for consistency and transparency in reporting. For further information on Nature Portfolio policies, see our Editorial Policies and the Editorial Policy Checklist.

Statistics

For all statistical analyses, confirm that the following items are present in the figure legend, table legend, main text, or Methods section.

n/a	Confirmed	
☐	☒	The exact sample size (*n*) for each experimental group/condition, given as a discrete number and unit of measurement
☐	☒	A statement on whether measurements were taken from distinct samples or whether the same sample was measured repeatedly
☐	☒	The statistical test(s) used AND whether they are one- or two-sided<br>*Only common tests should be described solely by name; describe more complex techniques in the Methods section.*
☐	☒	A description of all covariates tested
☐	☒	A description of any assumptions or corrections, such as tests of normality and adjustment for multiple comparisons
☐	☒	A full description of the statistical parameters including central tendency (e.g. means) or other basic estimates (e.g. regression coefficient) AND variation (e.g. standard deviation) or associated estimates of uncertainty (e.g. confidence intervals)
☐	☒	For null hypothesis testing, the test statistic (e.g. *F*, *t*, *r*) with confidence intervals, effect sizes, degrees of freedom and *P* value noted<br>*Give P values as exact values whenever suitable.*
☒	☐	For Bayesian analysis, information on the choice of priors and Markov chain Monte Carlo settings
☐	☒	For hierarchical and complex designs, identification of the appropriate level for tests and full reporting of outcomes
☐	☒	Estimates of effect sizes (e.g. Cohen's *d*, Pearson's *r*), indicating how they were calculated

Our web collection on statistics for biologists contains articles on many of the points above.

Software and code

Policy information about availability of computer code

Data collection	Inquisit 4 (2015) was used to collect the data
Data analysis	SPSS and R were used

For manuscripts utilizing custom algorithms or software that are central to the research but not yet described in published literature, software must be made available to editors and reviewers. We strongly encourage code deposition in a community repository (e.g. GitHub). See the Nature Portfolio guidelines for submitting code & software for further information.

Data

Policy information about availability of data

All manuscripts must include a data availability statement. This statement should provide the following information, where applicable:

- Accession codes, unique identifiers, or web links for publicly available datasets
- A description of any restrictions on data availability
- For clinical datasets or third party data, please ensure that the statement adheres to our policy

Studies were preregistered at Open Science Framework and data is posted there as well: https://osf.io/397tc/.

Research involving human participants, their data, or biological material

Policy information about studies with human participants or human data. See also policy information about sex, gender (identity/presentation), and sexual orientation and race, ethnicity and racism.

Reporting on sex and gender	Table 1 provides an overview of demographic data for each study, including children's gender as reported by parents
Reporting on race, ethnicity, or other socially relevant groupings	Table 1 provides an overview of demographic data for each study, including children's ethnicity as reported by parents
Population characteristics	Children were aged between 6 and 9 years
Recruitment	Children were recruited in a science museum or via after school day cares or an existing lab-database. Participation was voluntary.
Ethics oversight	Vrije Universiteit Amsterdam, Utrecht University

Note that full information on the approval of the study protocol must also be provided in the manuscript.

Field-specific reporting

Please select the one below that is the best fit for your research. If you are not sure, read the appropriate sections before making your selection.

☐ Life sciences ☒ Behavioural & social sciences ☐ Ecological, evolutionary & environmental sciences

For a reference copy of the document with all sections, see nature.com/documents/nr-reporting-summary-flat.pdf

Behavioural & social sciences study design

All studies must disclose on these points even when the disclosure is negative.

Study description	quantitative experimental
Research sample	Children, 6 to 9 years. Parental reports indicate that the majority of them identified as Dutch - and modal income and educational background are higher than the modal in the Netherlands.
Sampling strategy	Convenience sampling was used. Sample sizes were based on a priori power calculations based on effect sizes of previous studies and aiming to achieve 90% power (alpha is set at .05).
Data collection	Data collection took place at children's homes, after school day care and a science museum. the experiment was programmed in inquisit and all instructions given via pre-recorded audio. Experimenters were therefore blind to condition.
Timing	study 1: August 19 2019 to September 1 2019<br>study 2: January 9 2020 to July 23 2020<br>study 3: December 30 2021 to July 9 2022
Data exclusions	Study 1: no exclusions<br>Study 2: Data for six children was excluded because they were older than 9 years (2 10-year-olds, 4 11-year-olds; due to experimenter error). As preregistered, children were also excluded when they failed the memory check about targets' competency during an earlier quiz (n =14)<br>Study 3: Four children were excluded (3 failed the memory check and 1 did not finish).
Non-participation	No dropout other that those stated above
Randomization	children were randomly allocated to condition

Reporting for specific materials, systems and methods

We require information from authors about some types of materials, experimental systems and methods used in many studies. Here, indicate whether each material, system or method listed is relevant to your study. If you are not sure if a list item applies to your research, read the appropriate section before selecting a response.

Materials & experimental systems

n/a	Involved in the study
☒	☐ Antibodies
☒	☐ Eukaryotic cell lines
☒	☐ Palaeontology and archaeology
☒	☐ Animals and other organisms
☒	☐ Clinical data
☒	☐ Dual use research of concern
☒	☐ Plants

Methods

n/a	Involved in the study
☒	☐ ChIP-seq
☒	☐ Flow cytometry
☒	☐ MRI-based neuroimaging
